# Synergistic Enhancement of Photocatalytic H_2_O_2_ Production over Carbon Nitride Oxide/Biochar Composites

**DOI:** 10.3390/molecules30224323

**Published:** 2025-11-07

**Authors:** Ruolin Cheng, Yue Wang, Shijian Lu

**Affiliations:** 1 Jiangsu Key Laboratory of Coal-Based Greenhouse Gas Control and Utilization, Carbon Neutrality Institute, China University of Mining and Technology, Xuzhou 221008, China; 2 School of Chemical Engineering, China University of Mining and Technology, Xuzhou 221116, China

**Keywords:** photocatalysis, hydrogen peroxide, carbon nitride, carbonyl functionalization, biomass utilization

## Abstract

The green synthesis of hydrogen peroxide (H_2_O_2_) is crucial for sustainable chemical production, but pristine graphitic carbon nitride (g-C_3_N_4_) suffers from low H_2_O_2_ yield owing to limited visible light absorption and swift charge recombination. Herein, a novel metal-free carbon nitride oxide/biochar photocatalytic system (CNO-B) was developed via a simple low-temperature calcination without post-treatment. The synergistic effect of carbonyl functionalization and biochar integration significantly enhanced light harvesting capabilities and charge carrier separation efficiency, achieving an exceptional H_2_O_2_ production rate of 2483 μmol g^−1^ h^−1^ upon irradiation (five times higher compared with pure g-C_3_N_4_). This work provides valuable insights into minimalist synthesis strategies for designing functional materials and demonstrates a practical approach for valorizing biomass waste in sustainable photocatalytic applications.

## 1. Introduction

Hydrogen peroxide (H_2_O_2_) is a multifaceted oxidizing agent widely employed in diverse industrial sectors, including disinfection, bleaching, and chemical synthesis, owing to its environmentally benign decomposition products [1]. However, its current industrial-scale production predominantly relies on the anthraquinone process [2], which is energy-intensive, requires precious-metal catalysts, and generates substantial waste, thereby contradicting the principles of green chemistry [3]. In response, photocatalytic H_2_O_2_ synthesis has been a viable sustainable alternative, utilizing solar energy to drive simultaneous oxygen reduction (ORR) and water oxidation (WOR) reactions under ambient conditions [4,5,6].

Extensive study has concentrated on the advancement of efficient semiconductor photocatalysts for H_2_O_2_ production, encompassing metal oxides [7,8], metal sulfides [9,10], metal–organic frameworks (MOFs) [11], and covalent organic frameworks (COFs) [12]. Graphitic carbon nitride (g-C_3_N_4_) has been the subject of extensive research owing to its low cost, ease of synthesis, high chemical stability, and inherent ability to facilitate the ORR [13,14]. Nonetheless, pristine g-C_3_N_4_ is constrained by intrinsic limits, including restricted absorption of visible light, swift recombination of photogenerated electron-hole pairs, and diminished charge carrier mobility, which collectively hinder its photocatalytic efficiency [15].

To address these challenges, we developed a dual-modification strategy to construct a novel metal-free carbon nitride oxide/biochar photocatalytic system termed CNO-B. This approach integrates two key elements: (1) carbon nitride oxide containing carbonyl groups (C=O) was synthesized via a facile low-temperature calcination process, which requires no post-treatment or additional ligands, and (2) the incorporation of waste-derived biochar to form a composite material. The C=O groups function as effective electron traps, suppressing charge recombination to promote proton-coupled electron transfer reactions [16,17]. Concurrently, biochar extends the spectral absorption range into the visible region and improves interfacial electron transfer kinetics. The optimized CNO-B catalyst achieves an exceptional H_2_O_2_ generation rate of 2483 μmol g^−1^ h^−1^, which is 5 times greater than pristine g-C_3_N_4_. This work not only demonstrates a sustainable pathway for efficient H_2_O_2_ synthesis but also highlights the valorization of biomass waste, contributing to the advancement of green photocatalytic technologies.

## 2. Results and Discussion

**Figure 1:**
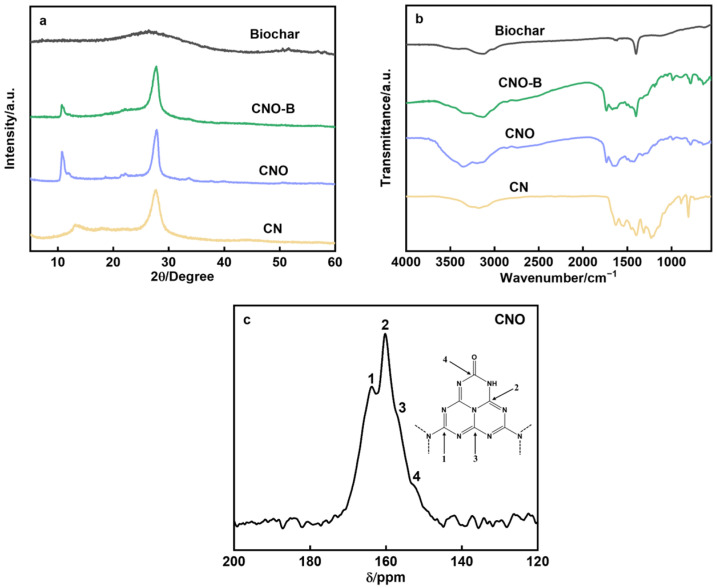
(**a**) XRD pattern and (**b**) FTIR pattern of biochar, CN, CNO, and CNO-B; (**c**) ^13^C NMR spectrum of CNO.

The samples’ structural and crystalline characteristics were investigated using XRD. As can be seen in Figure 1a, the characteristic diffraction peaks of CN are observed at 2θ = 12.8° and 27.5°. A shift in the (100) peak from 12.8° to 10.79° suggests structural rearrangement within the heptazine layers, resulting from the incorporation of oxygen-containing functional groups, which expand the inter-fragment spacing between the heptazine units [18]. New diffraction peaks emerge at 2θ = 21.5° in the modified CNO and CNO-B materials, attributed to partial oxidation of urea during synthesis that induces local distortion and reduces planarity of the heptazine layers [19]. The (002) peak’s constant location at 27.5° attests to the unaltered interlayer stacking. The XRD pattern of biochar shows a broad peak, and its presence in the CNO-B composite indicates successful integration of the two components.

FTIR spectra (Figure 1b) further reveals that CNO-B retains the core structure of CN, with absorption bands between 1000 and 1600 cm^−1^ corresponding to aromatic C-N heterocycles and a broad band at 3000–3500 cm^−1^ assigned to N-H/O-H stretches. A prominent peak at 1400 cm^−1^ in CNO-B confirms the presence of -OH groups [20], while new absorptions at 1740 cm^−1^ in both CNO and CNO-B are attributed to carbonyl (C=O) vibrations [21]. The synergistic modification through carbonyl functionalization and biochar incorporation is further reflected in a bathochromic shift in the s-triazine ring vibration from 810 cm^−1^ to 783 cm^−1^, indicating modulation of the electronic structure. Complementarily, solid-state ^13^C NMR spectroscopy (Figure 1c) confirms the local bonding environment of CNO. The dominant resonance at 160.2 ppm is attributed to N–C=N in the heptazine ring, while the signals at 163.8 and 156.8 ppm are assigned to N_2_C–NH_X_ and CN_3_, respectively. A further signal at 152.5 ppm, characteristic of C=O, evidences the successful grafting of oxygen-containing functionalities onto the framework.

XPS was employed to clarify the surface elemental composition and chemical states of the samples, complementing insights from XRD, FTIR, and NMR analyses. The survey spectra (Figure S1) validate the presence of C, N, and O in all materials.

**Figure 2:**
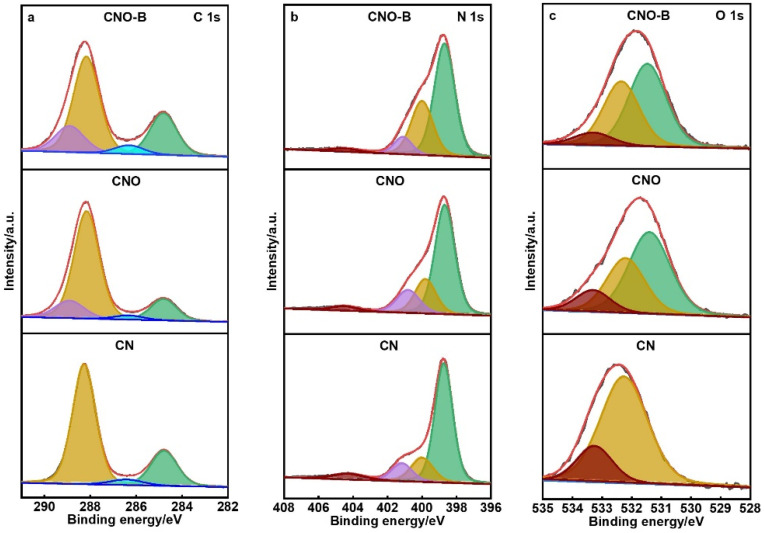
XPS spectra: (**a**) C 1s, (**b**) N 1s, and (**c**) O 1s of CN, CNO, and CNO-B.

High-resolution C 1s spectra (Figure 2a) display peaks at 284.8 eV (C=C), 286.6 eV (C-N), and 288.0 eV (N-C=N). Notably, both CNO and CNO-B show an additional peak at 289.0 eV, assigned to C=O bonds, which is absent in pristine CN [22]. Compared to CNO, the C 1s spectrum of CNO-B displays a slight positive binding energy shift along with a significant enhancement in C=O intensity, suggesting altered electronic environments likely induced by biochar incorporation. In the N 1s spectra (Figure 2b), all samples show signals at 398.6 eV (sp^2^ N in triazine rings), 399.9 eV (N-(C)_3_), and 401.0 eV (N-H). An increased peak area at 399.9 eV in CNO-B indicates that biochar modulates the electron density around nitrogen atoms [23]. Furthermore, the O 1s spectra (Figure 2c) reveal two common peaks at 532.2 eV and 533.3 eV, attributed to adsorbed H_2_O and O_2_ [24,25]. Critically, new peaks emerge at 531.4 eV in both CNO and CNO-B, corresponding to C=O species [17]. These XPS results collectively verify the successful incorporation of carbonyl groups in CNO and CNO-B and clearly differentiate them from unmodified CN, corroborating the effectiveness of the functionalization strategy.

**Figure 3:**
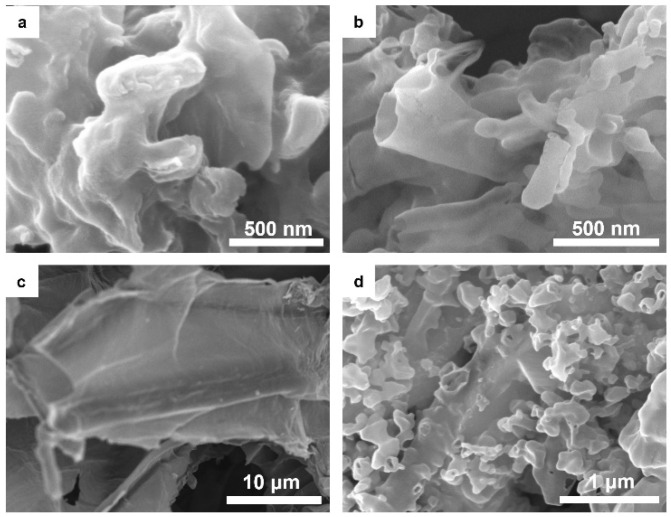
SEM images of (**a**) CN, (**b**) CNO, (**c**) Biochar, and (**d**) CNO-B.

Morphological analysis reveals that CN exhibits a disordered bulk structure with an even surface (Figure 3a), while CNO adopts an irregular rod-like structure (Figure 3b). Biochar displays a bulk band structure with a rough surface (Figure 3c) [26], and CNO is uniformly dispersed across the biochar surface, enhancing the surface roughness and porosity of the composites (Figure 3d and Figure S2) [27]. This close integration improves the photoactivity by facilitating the transfer of photogenerated carriers [20].

The wettability of the materials was evaluated by water contact angle measurements (Figure S3), employing the sessile drop method. The contact angles were determined by fitting the droplet profile with the Young-Laplace equation, with a measurement error of ±2° [28]. All samples demonstrated hydrophilicity, with contact angles of 36.5° for CN, 22.8° for CNO, and 32.2° for CNO-B, respectively. This trend suggests that the introduction of carbonyl groups significantly enhances surface hydrophilicity. Compared to CNO, the slightly larger contact angle of CNO-B is attributed to the intrinsic hydrophobicity of the incorporated biochar, which moderately counteracts the overall hydrophilicity.

**Figure 4:**
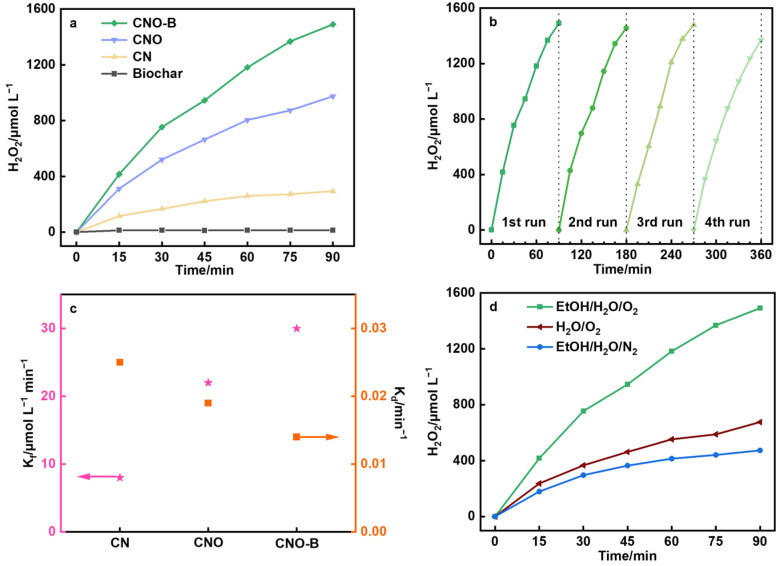
(**a**) Photocatalytic H_2_O_2_ yield of the samples (reaction conditions: 1 bar O_2_, 40 mg catalyst, 90 min, H_2_O:EtOH = 9:1, AM1.5 irradiation), (**b**) The yield variation in CNO-B during the 360 min reaction process in the reaction system, (**c**) H_2_O_2_ formation and decomposition constants on samples, and (**d**) H_2_O_2_ yield of CNO-B under different atmosphere/solution conditions.

The photocatalytic H_2_O_2_ production was evaluated under an oxygen atmosphere, with performance metrics unequivocally demonstrating the impact of sequential material modifications (Figure 4a). Biochar barely has H_2_O_2_ production activity, which is only 14 μmol L^−1^. The H_2_O_2_ yields for CNO-B, CNO, and CN were measured as 1490, 974, and 290 μmol L^−1^, respectively, establishing a clear performance hierarchy of CNO-B > CNO > CN. This progression highlights that the introduction of C=O groups in CNO and the further incorporation of biochar in CNO-B synergistically enhance photoactivity, with CNO-B achieving a yield nearly 5 times higher than pristine CN. CNO-B also delivers robust visible-light photoactivity, yielding 657 μmol L^−1^ H_2_O_2_ under λ ≥ 420 nm illumination (Figure S4). Compared to the recently reported works, CNO-B shows outstanding performance (Table S1). Moreover, CNO-B exhibits exceptional cycling stability, maintaining consistent performance over 4 reuse cycles without significant activity loss (Figure 4b). XRD analysis of CNO-B after the reaction confirmed the integrity of its structure (Figure S5).

From a kinetic perspective, the significantly enhanced H_2_O_2_ yield of CNO-B implies a substantially increased generation rate constant (K_f_) and a pronounced decrease in the decomposition rate constant (K_d_), which collectively attribute to CNO-B’s superior H_2_O_2_ production efficiency (Figure 4c) [29]. Control experiments further decode the origin of this enhancement (Figure 4d): in an N_2_ atmosphere, the H_2_O_2_ yield collapses to 473 μmol L^−1^, confirming that ORR is the dominant H_2_O_2_ source; in the absence of ethanol, it falls further to 675 μmol L^−1^, revealing its dual role as both a sacrificial agent for holes and a proton supplier for H_2_O_2_ formation [30].

Systematic physicochemical characterization was conducted to unravel the intrinsic factors governing the photocatalytic performance of CN, CNO, and CNO-B catalysts.

Firstly, depicted from N_2_ adsorption–desorption isotherms, the specific surface area of CN was 10 m^2^ g^−1^, indicating its inherently compact structure (Figure S6). Remarkably, CNO-B exhibited the greatest specific surface area of 36 m^2^ g^−1^, about double that of CNO (16 m^2^ g^−1^) and 3.6 times larger than CN, resulting in a more porous and accessible morphology.

**Figure 5:**
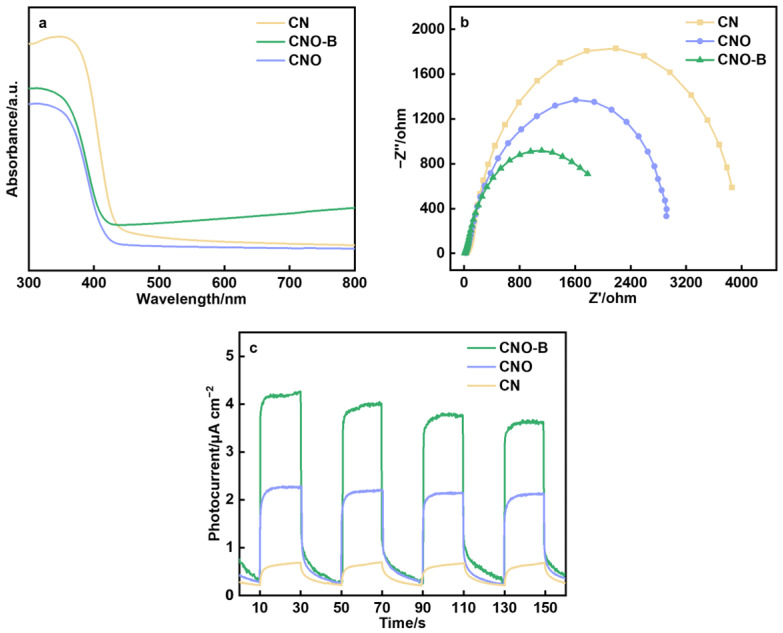
Physiochemical properties analysis: (**a**) UV-Vis absorption spectra, (**b**) EIS plots and (**c**) transient light response of the catalysts.

From the view of light utilization ability, though CNO exhibits a blue-shifted absorption edge compared to CN, the introduction of black biochar in CNO-B effectively compensates for this limitation (Figure 5a) [31]. CNO-B shows increased absorption in the 400–800 nm visible light range. Tauc plots (Figure S7a) were used to calculate the band gaps, which came out to be 2.86 eV for CN and 2.95 eV for CNO [32]. The valence band (VB) positions for CN and CNO, obtained from XPS valence band spectra (Figure S7b), were identified at 2.12 eV and 2.15 eV (vs. NHE), respectively. Consequently, the conduction band (CB) positions were determined to be −0.74 eV for CN and −0.80 eV (vs. NHE) for CNO. The comparable band edge positions imply that the enhanced photocatalytic H_2_O_2_ production performance of CNO stems from factors beyond band energetics.

EIS illustrate the charge transfer resistance through Nyquist plots (Figure 5b). Out of all the catalysts, CNO-B has the smallest semicircle radius, indicating the lowest charge transfer resistance. In accordance, under periodic illumination, CNO-B generates the highest photocurrent density, demonstrating a rapid and robust photo-response (Figure 5c). The enhanced separation and migration of photogenerated carriers substantially enhance photocatalytic activity. Collectively, these analyses establish that CNO-B achieves broadened light absorption, and superior charge carrier dynamics through the synergistic integration of multiple modification strategies.

**Figure 6:**
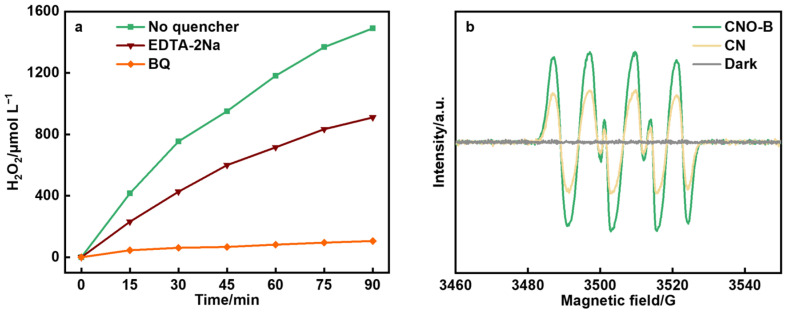
(**a**) Radical trapping experiments of CNO-B and (**b**) EPR analysis of the catalysts.

To unravel the photocatalytic reaction mechanism of H_2_O_2_ production over the catalysts, radical trapping experiments and EPR analysis were systematically employed. As shown in Figure 6a, to confirm the crucial role of photogenerated holes (h^+^) in the process, EDTA-2Na was introduced to the original system as a dedicated hole scavenger, resulting in a significant decrease in H_2_O_2_ production. Notably, the incorporation of BQ, a quencher of superoxide radicals, led to a drastic reduction in H_2_O_2_ production, clearly underscoring the essential function of O_2_^−^ in the catalytic cycle. Further validation of the generation of superoxide radicals (·O_2_^−^) was provided by EPR under irradiation (Figure 6b), which displayed a characteristic quintet signal pattern (1:1:1:1:1) for both CN and CNO-B, with a significantly stronger signal intensity for CNO-B [33]. No EPR signal observed in the absence of light, underscoring the photo-induced nature of the process.

A feasible mechanism is postulated based on the above data and literature report [34]. Under irradiation, electrons in the valence band of CNO-B are stimulated to move into the conduction band, creating electron-hole pairs (CNO-B + hν → e^−^ + h^+^). The superoxide radicals (O_2_ + e^−^ → ·O_2_^−^) are then created when the photogenerated electrons reduce adsorbed oxygen molecules. Simultaneously, the holes participate in proton-generating pathways: they oxidize ethanol to produce protons and acetaldehyde (h^+^ + CH_3_CH_2_OH → CH_3_CH_2_O + H^+^), and to a lesser extent, they facilitate water oxidation to release protons and oxygen (2h^+^ + H_2_O → ½O_2_ + 2H^+^). The generated ·O_2_^−^ radicals subsequently react with additional electrons and protons to yield hydrogen peroxide (·O_2_^−^ + e^−^ + 2H^+^ → H_2_O_2_). The efficient coupling of oxygen reduction with hole-driven proton supply is crucial for high H_2_O_2_ selectivity and yield, minimizing charge recombination and side reactions.

## 3. Materials and Methods

### 3.1. Chemicals and Reagents

Shanghai Macklin Biochemical Technology Co., Ltd. (Shanghai, China) was the supplier of Urea (98%), anhydrous ethanol (EtOH, ≥99.8%), melamine (99%), potassium iodide (KI, ≥99%), p-benzoquinone (BQ, 99%), potassium hydrogen phthalate (C_8_H_8_KO_4_, ≥99.8%), sodium sulfate (Na_2_SO_4_, 99%), disodium ethylenediamine-tetraacetate (EDTA-2Na, ≥95). Prior to usage, none of the reagents underwent further purification.

### 3.2. Synthesis of g-C_3_N_4_ (CN)

Pure graphitic carbon nitride (g-C_3_N_4_, CN) was prepared by thermal polymerization of melamine. Briefly, 3 g of melamine was subjected to heat treatment at 520 °C (heating rate: 5 °C per minute) for 3 h. The product obtained was washed and gathered.

### 3.3. Synthesis of Carbon Nitride Oxide/Biochar Composites (CNO-B)

In a standard synthesis, sawdust was directly pyrolyzed for 2 h at 750 °C in a tubular furnace with an inert environment, heating at a rate of 5 °C per minute, to create biochar. Subsequently, 100 mg of the obtained biochar was thoroughly mixed with 4 g of urea. The mixture was heated in a muffle furnace for 1 h from room temperature to 405 °C, at a pace of 5 °C per minute. The resultant gray substance was collected and pulverized into a fine powder, denoted as CNO-B. For comparison, pristine carbon nitride oxide (CNO) was prepared under identical thermal conditions using 4 g of urea alone without biochar addition.

### 3.4. Characterization

To acquire X-ray diffraction (XRD) patterns, a Bruker D8 Advance diffractometer (Bruker AXS GmbH, Karlsruhe, Germany) was used. A Thermo Fisher Nicolet iS50 spectrometer (Thermo Fisher Scientific Inc., Waltham, MA, USA) was used to perform Fourier transform infrared (FTIR) spectroscopy. The ^13^C nuclear magnetic resonance (^13^C NMR) spectrum was obtained using a Bruker Avance Neo 400WB spectrometer (Bruker BioSpin GmbH, Rheinstetten, Germany). Morphological features and elemental distribution were investigated by scanning electron microscopy (SEM) and energy-dispersive X-ray mapping using an FEI Quanta FEG 250 instrument (FEI Company, Hillsboro, OR, USA). Chemical states and surface composition were examined using X-ray photoelectron spectroscopy (XPS) on a Thermo ESCALAB 250Xi system (Thermo Fisher Scientific Inc., Waltham, MA, USA) employing Al Kα radiation; all binding energies were calibrated relative to the C 1s peak at 284.8 eV. Water contact angles were obtained by the sessile-drop method on a Sindin SDC-350KS (Dongguan SINDIN Precision Instrument Co., Ltd., Dongguan, China). UV–visible diffuse reflectance spectra were obtained using a Shimadzu UV-2600 spectrophotometer (Shimadzu Corporation, Kyoto, Japan). N_2_ adsorption–desorption analysis were performed at 77 K using a Micromeritics ASAP 2020 PLUS analyzer (Micromeritics Instrument Corporation, Norcross, GA, USA). Electron paramagnetic resonance (EPR) spectroscopy was carried out on a Bruker EMXplus spectrometer (Bruker BioSpin GmbH, Rheinstetten, Germany) to detect paramagnetic species.

### 3.5. Photocatalytic H_2_O_2_ Production

The photocatalytic generation of H_2_O_2_ was performed by mixing 40 mg of the catalyst in a combination of 90 mL deionized water and 10 mL anhydrous ethanol. A 300 W Xenon lamp (CEL-HXUV300) (Beijing China Education Au-light Co., Ltd., Beijing, China) equipped with an AM1.5 filter was used as the light source to simulate full-spectrum solar irradiation. The reaction temperature was sustained at 25 °C via a condenser. Before illumination, the suspension was continuously purged with oxygen and vigorously agitated for 30 min in the dark to achieve adsorption–desorption equilibrium between the catalyst and the solution.

Throughout the reaction, 1 mL of suspension was intermittently extracted and filtered. The quantification of H_2_O_2_ was carried out using an iodometric spectrophotometric method. Specifically, the filtered solution was diluted 40-fold, and 1 mL of the diluted sample was combined with 1 mL of KI (0.4 M) and 1 mL of C_8_H_5_KO_4_ (0.1 M). The generated H_2_O_2_ oxidized I^−^ to I_3_^−^, resulting in a distinct absorption peak at 350 nm. Following a 30 min reaction, the absorbance at 350 nm was recorded using a UV-vis spectrometer (Shimadzu Corporation, Kyoto, Japan), and the H_2_O_2_ concentration was determined based on a standard calibration curve.

### 3.6. Photoelectrochemical Analysis

A DH7000 electrochemical workstation (Jiangsu Donghua Analytical Instrument Co., Ltd., Taizhou, China) in a conventional three-electrode setup was used for photoelectrochemical evaluations. A platinum plate acted as the counter electrode, while a saturated Hg/HgO (SCE) functioned as the reference electrode. The working electrode was fabricated by applying 20 mg of the catalyst onto fluorine-doped tin oxide (FTO) glass, yielding a uniform film with a geometric area of 1 cm^2^. The electrolyte comprised 50 mL of a 0.2 M aqueous solution of Na_2_SO_4_. Electrochemical impedance spectroscopy (EIS) was conducted in darkness at an applied potential of −0.4 V. The transient photocurrent response was assessed under modulated light irradiation at a bias potential of 0.4 V.

## 4. Conclusions

In summary, a novel metal-free carbon nitride oxide/biochar (CNO-B) photocatalytic system was constructed through a straightforward low-temperature calcination to address the inherent limitations of pristine graphitic carbon nitride (g-C_3_N_4_) for sustainable H_2_O_2_ production. The synthesized material features carbonyl-rich carbon nitride oxide for enhanced charge separation, coupled with waste-derived biochar that extends light absorption and improves interfacial charge transfer. As a result, the optimized CNO-B catalyst achieves a superior H_2_O_2_ production rate of 2483 μmol g^−1^h^−1^, which is fivefold that of pure g-C_3_N_4_, alongside excellent cyclic stability. This work highlights the promise of combining defect engineering with carbon-based hybridization for advancing semiconductor photocatalysts and showcases a minimalist, sustainable route to valorize biomass waste into functional catalytic materials.

## Data Availability

The original contributions presented in this study are included in the article/Supplementary Material. Further inquiries can be directed to the corresponding author(s).

## Supplementary Materials

The following supporting information can be downloaded at https://www.mdpi.com/article/10.3390/molecules30224323/s1, Figure S1: XPS survey spectra; Figure S2: SEM image and the corresponding element mappings of CNO-B; Figure S3: Water contact angles; Figure S4: The effect of light wavelength on the photocatalytic H_2_O_2_ production performance of CNO-B; Figure S5: XRD pattern of CNO-B before and after the stability test; Table S1: Previously reported studies on photocatalytic H_2_O_2_ production; Figure S6: N_2_ adsorption–desorption isotherms; Figure S7: Tauc plots and XPS valence band spectra. Refs. [35,36,37,38,39,40,41,42,43] are cited in the Supplementary Materials.
