# Supplementary material for: Synergistic Enhancement of Photocatalytic H2O2 Production over Carbon Nitride Oxide/Biochar Composites
Source: Molecules. 2025 Nov 7;30(22):4323. doi: 10.3390/molecules30224323 (PMC12654964; doi:10.3390/molecules30224323)
Supplement: Supplementary file 1 [file molecules-30-04323-s001.zip › molecules-3949428-SI.pdf]

Supporting Information

Synergistic Enhancement of Photocatalytic H_2O_2 Production over Carbon Nitride Oxide/Biochar Composites

Ruolin Cheng [1,2,*], Yue Wang [2] and Shijian Lu [1,2,*]

[1] Jiangsu Key Laboratory of Coal-Based Greenhouse Gas Control and Utilization, Carbon Neutrality Institute, China University of Mining and Technology, Xuzhou 221008, China
[2] School of Chemical Engineering, China University of Mining and Technology, Xuzhou 221116, China
* Correspondence: ruolin.cheng@cumt.edu.cn (R.C.); lushijian@cumt.edu.cn (S.L.)

Academic Editor: Sergio Navalon

Received: 10 October 2025
Revised: 28 October 2025
Accepted: 5 November 2025
Published: 7 November 2025

Citation: Cheng, R.; Wang, Y.; Lu, S. Synergistic Enhancement of Photocatalytic H_2O_2 Production over Carbon Nitride Oxide/Biochar Composites. *Molecules* **2025**, *30*, x. https://doi.org/10.3390/xxxxx

Copyright: © 2025 by the authors. Submitted for possible open access publication under the terms and conditions of the Creative Commons Attribution (CC BY) license (https://creativecommons.org/licenses/by/4.0/).

Supplementary Figures and Tables

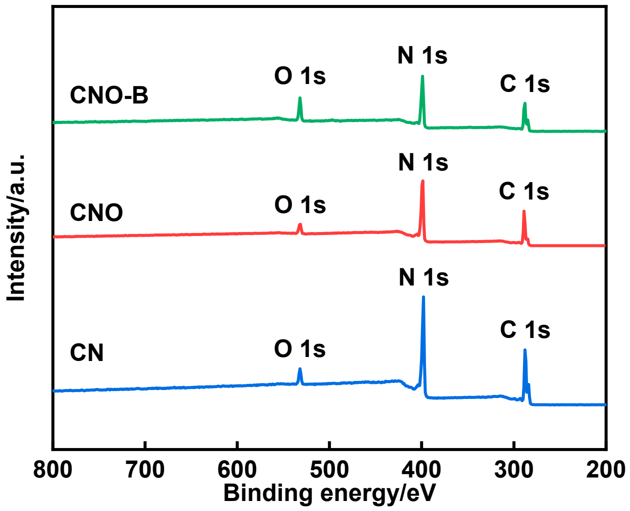

Figure S1. XPS survey spectra of the catalysts.

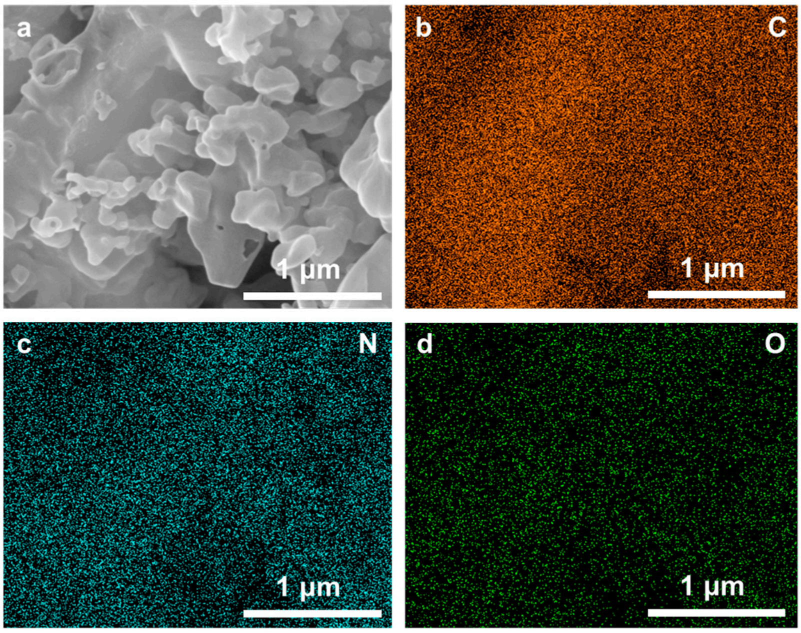

Figure S2. SEM image (a) and the corresponding element mappings (b-d) of CNO-B.

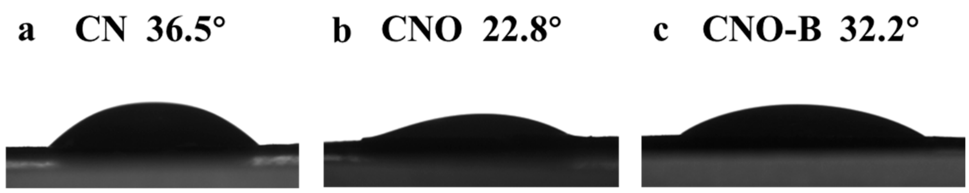

Figure S3. Water contact angles of (a) CN, (b) CNO, and (c) CNO-B.

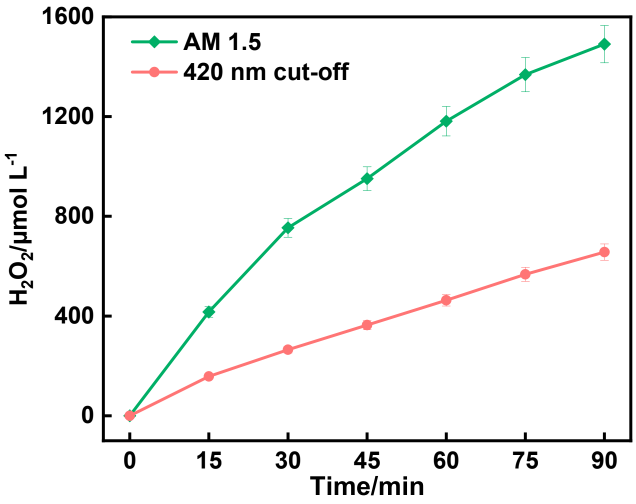

Figure S4. The effect of light wavelength on the photocatalytic H_2O_2 production performance of CNO-B.

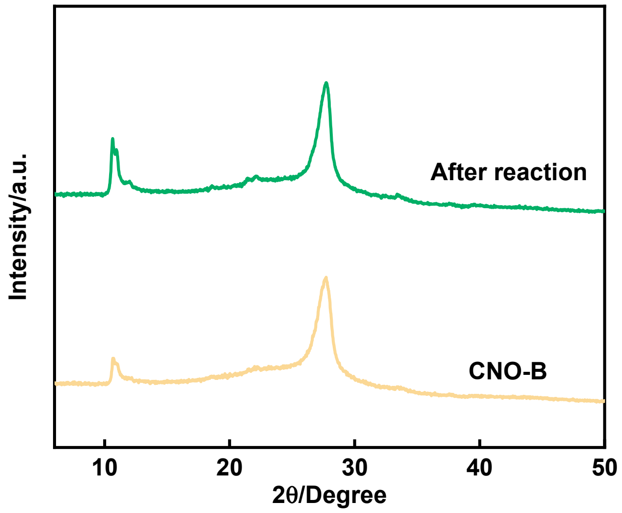

Figure S5. XRD pattern of CNO-B before and after the stability test

Table S1. Previously reported studies on photocatalytic H_2O_2 production.

Photocatalyst	Catalyst dosage/ mg	Solution	Light source	Time/ min	H_2O_2 yield/ μmol $g^{-1}h^{-1}$	Ref
g-C_3N_4/500-85 °C-3h	30	30 mL water	5 W LED	420	30	[1]
CN/HEC	30	30 mL water	300W Xe Lamp	60	66	[2]
CN-Au/$BiVO_4$	80	80 mL citrate buffer solution	420 nm LED	120	675	[3]
TA-CN-3	25	50 mLwater	300W Xe Lamp visible light	60	284	[4]
KLCN	50	50 mL water/IPA (Vw : Ve = 9 : 1)	300 W Xe Lamp	60	267	[5]
P-mMCNNS-5	50	50 mL water/EtOH (Vw : Ve = 9 : 1)	Sunlight	180	1083	[6]
MCN/SCN/CF	25	25 mL water	300 W Xe Lamp	60	137	[7]
m-CNNP	30	30 mLwater/IPA (Vw : Ve = 9 : 1)	300W Xe Lamp visible light	60	43	[8]
5%NiS_2/CN	3	60 mL water	300 W Xe Lamp	60	300	[9]
CNO-B	40	100 mL water/EtOH (Vw : Ve = 9 : 1)	300 W Xe Lamp	90	2483	This work

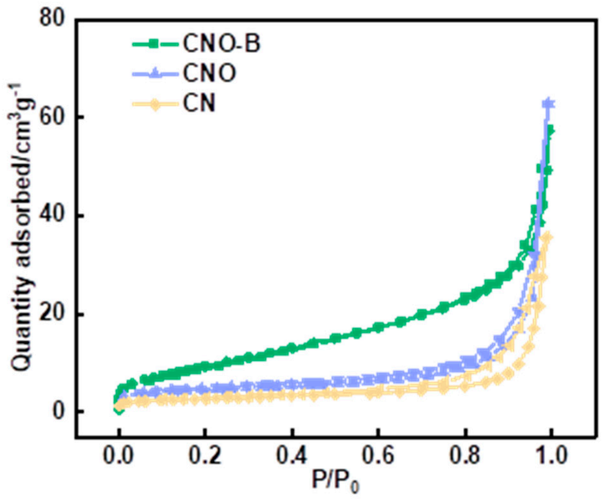

Figure S6. N_2 adsorption-desorption isotherms.

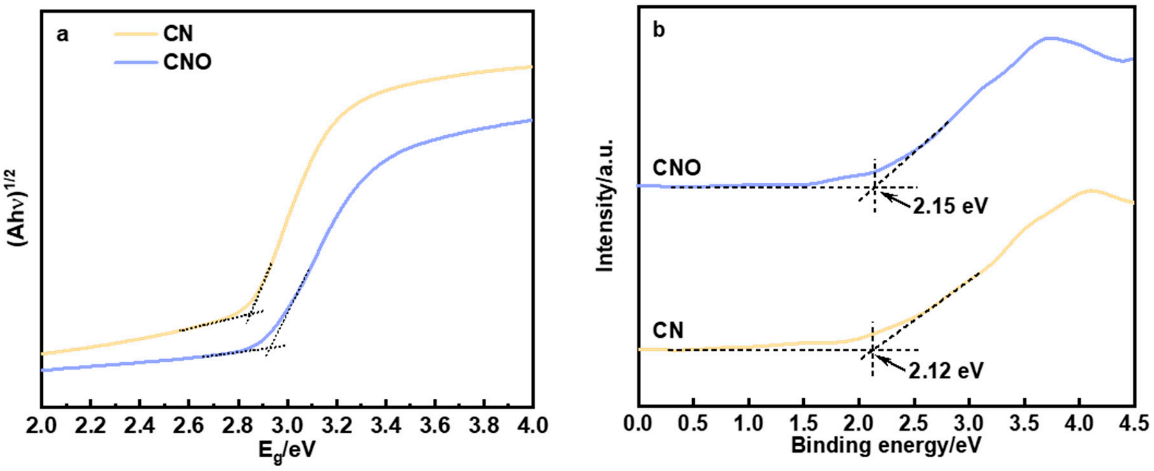

Figure S7. (a) Tauc plots, and (b) XPS valence band spectra of CN and CNO.

REFERENCES

1 Liu, B.; Du, J.; Ke, G.; Jia, B.; Huang, Y.; He, H.; Zhou, Y.; Zou, Z. Boosting O_2 Reduction and H_2O Dehydrogenation Kinetics: Surface N-Hydroxymethylation of g-C_3N_4 Photocatalysts for the Efficient Production of H_2O_2. *Adv. Funct. Mater.*, **2022**,*32*,2111125.

2 Zhang, P.; Zhang, J.; Wang, D.; Zhang, F.; Zhao, Y.T.; Yan, M.; Zheng, C.; Wang, Q.; Long, M.; Chen, C. Modification of g-C_3N_4 with hydroxyethyl cellulose as solid proton donor via hydrogen bond to enhance H_2O_2 production, *Appl. Catal. B*, **2022**,*318*,121749.

3 Shi, H.; Li, Y.; Wang, X.; Yu, H.; Yu, J. Selective modification of ultra-thin g-C_3N_4 nanosheets on the (110) facet of Au/$BiVO_4$ for boosting photocatalytic H_2O_2 production, *Appl. Catal. B*, **2021**,*297*,120414.

4 Shen, Y.; Shi, J.; Wang, Y.;Shi, Y.; Shan, P.; Zhang, S.; Hou, J.; Guo, F.; Li, C.; Shi, W. Incorporation of hydroxyl groups and π-rich electron domains into g-C_3N_4 framework for boosted sacrificial agent-free photocatalytic H_2O_2 production, *Chem. Eng. J.*,**2024**,*498*,155744.

5 Xu, Y.; Wang, G.; Li, W.; Yuan, Z.; Si, C. Broad-spectrum responsive lignin-KOH co-modified graphitic carbon nitride for synergetic photocatalytic H_2O_2 production via carbon-ring embedding and defect engineering, *Chem. Eng. J.*, **2025**,*503*,158655.

6 Zhou, L.; Feng, J.; Qiu, B.; Zhou, Y.; Lei, J.; Xing, M.; Wang, L.; Zhou, Y.; Liu, Y.; Zhang, J. Ultrathin g-C_3N_4 nanosheet with hierarchical pores and desirable energy band for highly efficient H_2O_2 production, *Appl. Catal. B*, **2020**,*267*,118396.

7 Zhou, J.; Shan, T.; Zhang, F.; Boury, B.; Huang, L.; Yang, Y.; Liao, G.; Xiao, H.; Chen, L. A Novel Dual-Channel Carbon Nitride Homojunction with Nanofibrous Carbon for Significantly Boosting Photocatalytic Hydrogen Peroxide Production, *Adv. Fiber Mater.*, **2024**,*6*,387-400.

8 Liu, W.; Song, C.; Kou, M.; Wang, Y.; Deng, Y.; Shimada, T.; Ye, L. Fabrication of ultra-thin g-C_3N_4 nanoplates for efficient visible-light photocatalytic H_2O_2 production via two-electron oxygen reduction, *Chem. Eng. J.*, **2021**,*425*,130615.

9 Zhang, Z.; Chen, C.; Tayyab, M.; Wei, Z.; Zheng, X.; Shangguan, W.; Zhang, S.; Chen, S.; Meng, S. Regulating electron-hole pairs of g-C_3N_4 efficiently separated and fully utilized for photosynthesis of H_2O_2 under visible light, *Chem. Eng. J.*, **2025**,*509*,161409.
